# Treatment of oligo-metastatic pancreatic ductal adenocarcinoma to the liver: is there a role for surgery? A narrative review

**DOI:** 10.1097/JS9.0000000000001665

**Published:** 2024-05-29

**Authors:** Felice Giuliante, Elena Panettieri, Andrea Campisi, Alessandro Coppola, Maria Vellone, Agostino M. De Rose, Francesco Ardito

**Affiliations:** a Hepatobiliary Surgery, Fondazione Policlinico Universitario Agostino Gemelli ‘IRCCS’, Università Cattolica del Sacro Cuore; b Department of Surgical Oncology, The University of Texas MD Anderson Cancer Center, Houston, Texas, USA; c Department of Surgery, Sapienza University of Rome, Rome, Italy

**Keywords:** liver metastases, liver resection, metastatic pancreatic adenocarcinoma, pancreatic ductal adenocarcinoma, perioperative chemotherapy

## Abstract

Pancreatic ductal adenocarcinoma (PDAC) is a prognostically unfavorable malignancy that presents with distant metastases at the time of diagnosis in half of patients. Even if patients with metastatic PDAC have not been traditionally considered candidates for surgery, an increasing number of researchers have been investigating the efficacy of surgical treatment for patients with liver-only oligometastases from PDAC, showing promising results in extremely selected patients, mainly with metachronous metastases after perioperative chemotherapy. Nevertheless, a standardized definition of oligometastatic disease should be adopted and additional investigations focusing on the role of perioperative chemotherapy and tumor biology are warranted to reliably assess the role of resection for PDAC metastatic to the liver.

## Introduction

Highlights

- Patients with liver metastases from pancreatic ductal adenocarcinoma typically face a poor prognosis and are not traditionally considered as surgical candidates.
- A standardized definition of oligometastatic disease should be adopted and the role of perioperative chemotherapy should be further investigated in this population.
- Nonetheless, the present review suggests that surgical treatment of patients with oligometastatic pancreatic ductal adenocarcinoma to the liver can be considered a safe procedure and potentially beneficial in terms of survival, especially in carefully selected cases.
- In particular, it seems that response to preoperative chemotherapy or use of postoperative chemotherapy, along with limited liver metastatic disease, could offer higher possibility of longer survival.

Pancreatic ductal adenocarcinoma (PDAC) is a prognostically unfavorable malignancy, with an overall 5-year survival rate of 10%, and, with a steadily increasing incidence, is becoming one of the leading causes of cancer-related death globally^1,2^. Despite recent advances in perioperative combination therapies, surgery - including resection of the primary pancreatic tumor and regional lymph nodes - remains the only potentially curative treatment^3^. However, 50–55% of PDAC patients present with metastatic disease at the time of diagnosis, deeming curative treatment unfeasible^4,5^. Moreover, radiologically occult metastases might be detected during laparotomy or staging laparoscopy in patients who are candidates for radical surgery, despite not being visible at preoperative imaging studies. The occurrence of these findings, such as liver metastases and peritoneal dissemination, ranges between 14 and 30% among these patients^6^.

Notably, three-quarters of patients undergoing curative-intent surgery combined with perioperative therapy for PDAC experience disease recurrence within 2 years^7,8^. In this context, the aggressive behavior of this tumor has traditionally discouraged surgery for metastatic patients since pancreatectomy was not associated with a survival benefit in this population^9,10^. Nevertheless, an increasing number of researchers have been investigating the efficacy of surgical treatment for patients with liver-only oligometastases from PDAC, showing promising results in extremely selected cases^11^. In this setting, the debate revolves around the effectiveness of surgical treatment in different kind of patients, with synchronous or metachronous liver metastases from PDAC. In both cases, tumor biology needs to be strongly considered and assessed through clinical observation and response to systemic treatment.

Aim of this review is to summarize the available evidence on the role of surgery for metastatic PDAC to the liver.

## Indication to pancreatic adenocarcinoma resection, advancements in perioperative therapy, and current guidelines

PDAC resectability is mainly based on the radiologic anatomical involvement of the celiac axis, the common hepatic artery, the superior mesenteric artery, the superior mesenteric vein, and the portal vein. The extent of the circumferential tumor-vessel interface and the ability to perform vascular reconstruction determine the attribution of PDAC to one of the following categories: potentially resectable, borderline resectable, and unresectable^12^.

Nevertheless, radiologic criteria alone are not enough to guide therapeutic decision-making, and tumor biology and patient physiology should be considered as well. In particular, tumor aggressiveness is characterized by the presence of distant metastases and/or regional lymphadenopathy and CA19-9 levels^13,14^.

Current guidelines recommend upfront PDAC resection when the following criteria are met: no clinical evidence for metastatic disease, a performance status and comorbidity profile appropriate for a major abdominal operation, no radiographic interface between primary tumor and mesenteric vasculature on high-definition cross-sectional imaging, and an acceptable CA 19-9 level (in absence of jaundice) suggestive of localized disease^15^.

Recognizing PDAC dismal prognosis even after optimal surgery, the utilization of preoperative therapy for potentially resectable PDAC has become increasingly popular, aiming at identifying patients with favorable tumor biology and obtaining systemic disease control.

This approach seems particularly reasonable since the high rate of complications after pancreatic surgery often delays the start of adjuvant treatment^14,16,17^. According to the ASCO Guidelines for potentially curable PDAC, preoperative therapy should be offered when: imaging is suspect – but not diagnostic – for extrahepatic disease, performance status is not optimal but could be reversed, there is some tumor-vascular interface, and CA 19-9 levels are elevated (in absence of jaundice)^15^. In this context, neoadjuvant treatment might help downsizing or reducing anatomical extent of borderline resectable PDAC and to select patients with favorable tumoral biology with the goal of allowing resection.

The ESPAC5 randomized clinical trial aimed at establishing the feasibility and efficacy of three different types of short-course neoadjuvant therapy compared with immediate surgery in borderline resectable PDAC. Neoadjuvant therapy showed a significant survival benefit compared with immediate surgery. In particular, 1-year overall survival (OS) was 39% for immediate surgery, 78% for gemcitabine plus capecitabine, 84% for FOLFIRINOX, and 60% for capecitabine-based chemoradiotherapy (*P*=0.003)^18^.

Primary resection followed by adjuvant therapy with FOLFIRINOX – or gemcitabine plus capecitabine or gemcitabine monotherapy as an alternative – has also been proposed as a treatment modality able to improve survival outcomes in localized PDAC when preoperative chemotherapy is not administered^19,20^.

For the time being, systemic therapy is the treatment of choice for metastatic PDAC and germline and somatic testing is recommended to select therapy, incusing targeted agents. No currently available guidelines mention resection as an option for patients with oligometastatic PDAC^21,22^. Nevertheless, several centers have published their series of resection for PDAC liver metastases, and the following paragraphs illustrate their experiences.

## Surgical treatment of pancreatic adenocarcinoma with synchronous liver metastases

The efficacy of surgical treatment combined with chemotherapy or chemoradiation compared to systemic palliative treatment alone for patients with oligometastatic PDAC to the liver is currently a subject of significant debate and conflicting results have been presented.

In 2016, Tachezy *et al*.^23^ conducted a multicenter study involving six European pancreatic surgery centers, retrospectively collecting data from 69 PDAC patients with synchronous hepatic metastases. These patients underwent pancreatic and simultaneous hepatic resection of a median of 2 (range: 1–11) liver metastases, including pancreatoduodenectomy in 43% of cases. Preoperative and postoperative chemotherapy were administered in 13 and 81% of patients, respectively. Resected patients showed a higher median OS compared to patients treated with conventional medical therapy (14 months vs. 8 months, *P*<0.001), despite a 58% R0 rate for the primary tumor and a 68% postoperative morbidity rate.

Shi *et al*.^24^ identified 30 patients with PDAC metastatic to the liver undergoing simultaneous pancreatic resection and hepatectomy, achieving a median OS of 15.7 months. Interestingly, liver metastases were detected preoperatively in only 50%, being the other 50% discovered at operation. In this study, patients undergoing combined surgical treatment and those without liver metastases undergoing pancreatic resection only for PDAC showed similar survival (15.7 months vs. 16.9 months, *P*=0.085) and had similar rates of postoperative pancreatic fistula (*P*=0.072).

Hackert *et al*.^25^ collected prospective data from 128 stage IV PDAC patients with liver or regional lymph node metastases who underwent pancreatic surgical resection and hepatic or aorto-caval lymph nodes resection. Among the 62 patients who underwent simultaneous resection of PDAC and liver metastases, the median survival was 10.6 months, with a postoperative morbidity rate of 45%. In this study, 96.4% of patients had ≤3 liver metastases and no data about use of chemotherapy was reported.

Yang *et al*.^26^ gathered 48 patients with liver-metastatic PDAC who underwent synchronous resection of the primary tumor and liver metastases, including 25 patients (52.1%) with nonoligometastatic disease, defined as: (i) metastases potentially extirpated by surgery or surgery combined with radiofrequency ablation (RFA), (ii) >3 metastases, (iii) metastases requiring venous or multivisceral resection. The median survival of the entire group was 7.8 months but, interestingly, resected patients with oligometastatic liver metastases had longer OS compared to non-oligometastatic resected patients (16.1 vs. 6.4 months, *P*=0.02), patients receiving systemic preoperative chemotherapy (16.1 months vs. 7.6 months, *P*=0.02), or palliative treatment (16.1 vs. 4.3 months; *P*<0.001).

Pausch *et al*.^27^ investigated the potential benefits of surgical treatment in patients with oligometastatic PDAC to the liver and sought to identify key prognostic factors in this population. They extracted data from 259 patients who underwent surgical resection for oligometastatic PDAC from the population-based Surveillance, Epidemiology, and End Results database (SEER). Surgical treatment for these patients resulted in a survival increase of 5–10 months. Multivariate analysis revealed that chemotherapy was a favorable prognostic factor, while advanced age and grade 3–4 tumor differentiation were unfavorable prognostic factors. Notably, 67.2% of patients in this series received systemic treatment, with no details on the timing of its administration. Furthermore, no information regarding the extent of liver involvement or postoperative outcomes was available.

Promising results were reported by Hamad *et al*.^28^, who queried the National Cancer Database (NCDB) obtaining 47 785 cases of stage IV PDAC with isolated liver metastases, 137 of which underwent simultaneous pancreatic and hepatic resection. Their results suggest that pancreatic resection combined with systemic therapy in case of stage IV PDAC with synchronous isolated liver metastases might offer, in selected cases, improved OS compared to palliative chemotherapy or chemoradiation alone. Furthermore, after propensity score matching, patients undergoing resection of the primary tumor and simultaneous hepatectomy along with systemic therapy had longer median OS (15.6 months vs. 8.1 months) compared to patients treated solely with medical therapy (*P*<0.001).

On the other hand, in a study involving nine patients with synchronous PDAC liver metastases, Dünschede *et al*.^29^ did not recommend combining pancreatic and liver resection, as surgical treatment did not seem to provide better survival than medical treatment with gemcitabine (8 vs. 11 months). In this small series, the rate of associated major liver resection was 33.3%.

Klein *et al*.^30^ compared postoperative morbidity and mortality in 22 patients with PDAC and incidental liver metastases who underwent simultaneous resection of the primary tumor and hepatic resection with that of 22 nonmetastatic PDAC patients undergoing pancreatic resection only. Considering that the combined-surgery group had a 32% R0 rate, survival was significantly higher in the population undergoing pancreatic resection only (7.5 months vs. 14.4 months; *P*=0.15). Notably, no information was available regarding liver metastases characteristics and type of liver resection. Nevertheless, since all the liver nodules were detected intraoperatively, it might be assumed that no major/anatomical resections were performed.

Zanini *et al*.^31^ focused on the timing of PDAC hepatic metastases diagnosis. In their case series, 11 patients underwent resection of the primary tumor combined with liver resection, while four patients underwent delayed hepatic resection for metachronous PDAC liver metastases. Liver metastases were identified intraoperatively in 8/11 patients of the simultaneous resection group. The median survival of the first group was 8.3 months, compared to 11.4 months in the second group (*P*=0.038), suggesting the timing of metastases onset as a potential prognostic factor for survival following surgery (Table 1).

**Table 1:** A summary of the articles describing outcomes of simultaneous resection of PDAC and liver metastases.

Author	Year	Country	No. of patients	Preoperative treatment	No. of liver metastases	Associated PD	Major hepatectomy	Postoperative morbidity	Postoperative mortality	R0	Postoperative treatment	OS
Dünschede *et al*.^29^	2010	Germany	9	–	Median 3; range 1–5	33.3%	33.3%	33%	0%	89%	–	8 months
Klein *et al*.^30^	2012	Germany	22	–	–	73%	–	18% ^b^	0%	32%	–	7.5 months
Zanini *et al*.^31^	2015	Italy	11	–	Median 2; range 1–3	54.5%	0%	54.5%	0%	47% pancreas 100% liver ^e^	–	8.3 months
Tachezy *et al*.^23^	2016	Germany, Italy, France, Greece	69	13%	Median 2; range 1–11	43%	–	68%	1.4%	58%	81%	14 months
Shi *et al*.^24^	2016	China	30	–	–	36.7%	–	–	0%	–	–	15.7 months
Hackert *et al*.^25^	2017	Germany	62	–	≤3 (96.4% of patients) ^a^	42.3% ^a^	1.6%	45%	1.6%	–	–	10.6 months
Yang *et al*.^26^	2020	China	48	27.1%	–	41.7%	2.1%	14.6%	4.2%	100%	–	7.8 months (16.1 months among oligometastatic patients)
Pausch *et al*.^27^	2022	Germany	259	67.2% ^b^	–	63.4% ^c^	–	–	–	–	67.2% ^b^	8–12 months
Hamad *et al*.^28^	2022	United States	137	67.2% ^b^	–	–	–	–	–	–	67.2% ^b^	15.6 months

OS, overall survival; PD, pancreatoduodenectomy; PDAC, pancreatic ductal adenocarcinoma.

a The study includes patients undergoing synchronous and metachronous resection of PDAC liver metastases. This percentage refers to the combined cohort (85 patients).

b Data refers to chemotherapy, with no details on the timing of treatment.

c Data refers to tumor localization in the pancreatic head.

d Clavien grade ≥3.

^e^ R1 resection rate refers to pancreatic resection in the entire cohort of 15 patients. An R0 resection was achieved for all liver resections.

## Surgical treatment of PDAC with metachronous liver metastases

The efficacy of surgical treatment for metachronous hepatic metastases from PDAC remains a subject of controversy, but it may represent a safe and effective therapeutic approach for carefully selected patients.

In contrast to the outcomes of patients undergoing simultaneous resection of PDAC and hepatic metastases from PDAC, in the Dünschede *et al*.’s study^29^, which also included four cases of PDAC with metachronous hepatic metastases, it appears that delayed hepatic resection may improve survival in selected patients. In particular, after comparison with five patients treated with gemcitabine only, resection of a median 1.75 metachronous liver metastases led to a median survival of 31 months vs. 11 months. The median interval of diagnosis of liver metastases from resection of the primary was 9 months (7–13 months) in the resection group and 6 months (2–11 months) in the gemcitabine group.

In the series reported by Hackert *et al*.^25^, 23 cases of PDAC with isolated metachronous liver metastases were identified. With a mean interval between pancreatic resection and hepatic resection of 18.4 months, patients achieved a median survival of 14.8 months, compared to the expected median 6–10 months of life expectancy with systemic treatment.

Schwarz *et al*.^32^ conducted a multicenter study including 25 patients, with a mean age of 63.8 years, who underwent hepatic resection for metachronous PDAC liver metastases. None of these patients died within 30 days of surgery, and three patients developed a Grade IIIa complication according to Clavien–Dindo^33^. This treatment approach demonstrated a significant increase in survival compared to palliative chemotherapy (36.8 months vs. 9.2 months, *P*=0.007). Notably, 88% of patients received postoperative systemic treatment.

More recently, Shibata *et al*.^34^ identified 35 patients with one single metachronous hepatic metastasis, and only 10 were selected for hepatic resection based on their institutional inclusion criteria. These criteria include (i) having a solitary tumor, (ii) demonstrating effective tumor control under systemic chemotherapy for more than 6 months (indicated by a return to normal tumor marker levels and stable tumor size on CT imaging, signifying a more controlled disease), and (iii) maintaining sufficient liver remnant function. By applying these selection criteria, the median survival time in the liver resection group (*n*=10) was significantly better than that in the nonliver resection group (*n*=25) (57.6 months vs. 20.1 months, *P*<0.001). Additionally, a multivariate analysis revealed that having multiple tumors [hazard ratio (HR), 2.26; 95% CI: 1.30–3.94; *P*=0.004] and not undergoing liver resection (HR, 6.90; 95% CI: 2.23–21.3; *P*<0.001) were independent risk factors for a poor prognosis (Table 2).

**Table 2:** A summary of the articles describing the surgical treatment for PDAC with metachronous hepatic oligometastases.

Author	Year	Country	No. of patients	Preoperative treatment	No. of liver metastases	Diagnostic interval from PDAC resection	Major hepatecomy	Postoperative treatment	OS
Dünschede *et al*.^29^	2010	Germany	4	–	Median 1.75; range 1–2	9 months	0%	–	31 months
Zanini *et al*.^31^	2015	Italy	4	–	1	9 months	25%	–	11.4 months
Hackert *et al*.^25^	2017	Germany	23	–	≤3 (96.4% of patients)^a^	–	26.1%	–	14.8 months
Schwarz *et al*.^32^	2020	Austria	25	**–**	Median 1; range 1-2	17.8 months	8%	88%	36.8 months
Shibata *et al*.^34^	2023	Japan	10	100%	1	10.7 months^b^	20%	–	57.6 months

OS, overall survival; PDAC, pancreatic ductal adenocarcinoma.

a The study includes patients undergoing synchronous and metachronous resection of PDAC liver metastases. This percentage refers to the combined cohort (85 patients).

b Recurrence-free survival refers to all patients included in the study (both resected and unresected) and to all sites of recurrence.

At the Fondazione Policlinico Universitario A. Gemelli ‘IRCCS’, resection of PDAC liver metastases is only considered in case of metachronous presentation. From 2000 to 2022, seven patients have been resected (unpublished data): five were males, with a median age of 61 years [interquartile range (IQR): 58–76]. Median interval from PDAC resection and liver metastases diagnosis was 13 months (IQR: 7–22). Preoperative chemotherapy was administered in three cases and two patients required a major hepatectomy. The median number of resected liver metastases was 1 (IQR: 1–3), with a median maximum size of 2.3 cm (IQR: 2–2.5). Median OS was 15.9 months.

## Discussion

Liver metastases are common in patients affected by PDAC and often associated with a worse prognosis compared to pulmonary or local recurrence, especially when multiple metastases involving several organs are detected, further diminishing long-term outcomes^34^. In this setting, it is crucial to treat this population in a multidisciplinary context to optimize results and stratify prognosis and therapeutic options, establishing criteria for selecting patients potentially amenable of surgical treatment.

In 1997, Takada *et al*.^35^ were the first to compare outcomes of patients with PDAC and hepatic metastases who underwent radical surgery or palliative interventions. In their study, 11 patients underwent pancreatoduodenectomy and partial hepatectomy, while 22 patients underwent palliative surgical bypass procedures. The median survival of these groups was comparable (6 months vs. 4 months, respectively). Nowadays, due to advancements in medical therapy, patients with metastatic PDAC are typically treated with palliative chemotherapy, providing a median life expectancy of 6–10 months^27^. However, in the past decade, several studies have shown that surgical treatment for PDAC with isolated liver metastases can be a valid therapeutic option in carefully selected patients.

In this context, it is important to highlight that there is lack of a standardized definition for oligometastatic disease, resulting in high heterogeneity in reporting results. A recent review on the definition and management of intra-abdominal metachronous oligometastatic PDAC demonstrated that, in 34 identified studies, 59% did not include a definition of oligometastatic disease and, of the 14 articles reporting a definition, none were the same^36^. This issue seems to be confirmed by the present review of the literature, particularly in the setting of synchronous PDAC liver metastases. More specifically, only four out of nine papers^23,25,29,31^ included details on the number of synchronous metastases resected. Additionally, three studies^24,30,31^ explained that 50–100% of the liver metastases were detected intraoperatively. In this scenario, it is reasonable to deduce that hepatic involvement was frequently an intraoperative finding and that wedge resections were mostly performed, as described by Klein *et al*.^30^.

An expert panel at the 2022 Joint Meeting of the International Association of Pancreatology (IAP) and the Japan Pancreas Society (JPS)^37^ described two major approaches on surgical resection for metastatic PDAC, namely the surgery-first and chemotherapy-first approaches. They summarized indications for the surgery-first approach – where patients underwent simultaneous resection of the primary tumor and liver metastases – based on findings from 11 papers: liver metastases have to be categorized as oligometastases or occult metastases (that were not identified at preoperative imaging), no distant metastases, other than liver metastases, have to be present, and margin-negative resection should be anticipated without posing undue surgical risks. The chemotherapy-first approach, on the other hand, consists in resecting the primary tumor after distant metastases have either disappeared or shown significant shrinkage on imaging studies following preoperative treatment for a certain period of time. This approach is commonly referred to as ‘conversion surgery’.

As highlighted in the present review, simultaneous resection of PDAC and liver metastases is associated with acceptable operative risk and improved survival in the majority of the reported articles, with a median survival ranging from 7.5 to 16.1 months^23–31^. Nevertheless, only four out of nine papers reported information regarding perioperative chemotherapy, that was administered in an extremely inhomogeneous rate of patients (13–81%). It is important to note how the role of neoadjuvant chemotherapy in the treatment of PDAC is relatively recent and still matter of debate, even if it is commonly considered as the first treatment in patients with PDAC metastatic to the liver^15,21,38^.

When focusing on metachronous PDAC liver metastases, with a recurrence-free survival from PDAC resection ranging from 9 to 17.8 months, survival after liver resection varied from 11.4 to 57.6 months^25,29,31,32,34^. Such longer survivals and the fact that most patients had solitary metastases, support the idea that the analyzed patients had a more indolent and biologically favorable disease compared to those with synchronous metastases. Interestingly, longest survivals were showed in the series were the majority of patients received perioperative chemotherapy^32,34^.

While this scenario seems encouraging, the scarcity of reports focusing on this topic, heterogeneity of reported data, and the small number of patients presented in the published series require caution in analyzing results. Additional investigations – ideally randomized controlled trials or multicenter registers – are warranted to reliably assess the role of resection for PDAC metastatic to the liver. In particular, more evidence on the role of chemotherapy in controlling disease in patients with PDAC oligometastatic to the liver is necessary. Notably, the spreading utilization of tools such as radiomics, genomic sequencing, transcriptional analysis, and microbiome testing will help gaining better understanding of tumor biology and to more appropriately select patients for surgery^14^.

Hopefully, once more substantial data confirming survival improvement and safety of surgical treatment of PDAC metastatic to the liver will be available, the leading experts of the field might be able to design specific guidelines.

## Conclusion

Patients with liver metastases from PDAC typically face a poor prognosis. A standardized definition of oligometastatic disease should be adopted and the role of perioperative chemotherapy should be further investigated in this population. Nonetheless, the present review suggests that surgical treatment of patients with oligometastatic PDAC to the liver can be considered a safe procedure and potentially beneficial in terms of survival, especially in carefully selected cases. In particular, it seems that response to preoperative chemotherapy or use of postoperative chemotherapy, along with limited liver metastatic disease, could offer higher possibility of longer survival.

## Ethical approval

Ethical approval was not required for the present study.

## Consent

Not applicable.

## Source of funding

No fundings were received to write the present paper.

## Author contribution

E.P., A.C., A.C., and M.V.: review of literature and paper writing; E.P., A.M.D.R., F.A., and F.G.: study concept and design.

## Conflicts of interest disclosure

The authors have no conflict of interest.

## Research registration unique identifying number (UIN)

1. Name of the registry: not applicable.
2. Unique identifying number or registration ID: not applicable.
3. Hyperlink to your specific registration (must be publicly accessible and will be checked): not applicable.

## Guarantor

Felice Giuliante.

## Data availability statement

The present paper is a narrative review of the literature and no patients’ data were included.

## Provenance and peer review

This was a paper invited by Prof. Roberto Coppola.
